# Development of an upper limb muscle strength rehabilitation assessment system using particle swarm optimisation

**DOI:** 10.3389/fbioe.2025.1619411

**Published:** 2025-07-09

**Authors:** Chuangan Zhou, Siqi Wang, Meiyi Wu, Wei Lai, Junyu Yao, Xingyue Gou, Hui Ye, Jun Yi, Dong Cao

**Affiliations:** ^1^ School of medical information engineering, Guangzhou University of Chinese Medicine, Guangzhou, Guangdong, China; ^2^ College of Business, City University of Hong Kong, Hong Kong SAR, China; ^3^ School of Medical Information Engineering, Guangdong Pharmaceutical University, Guangzhou, Guangdong, China

**Keywords:** upper limb movement disorders, surface electromyographic signals, feature extraction, regression prediction, feature importance, muscle strength assessment PSO-optimized upper limb rehabilitation strength assessment

## Abstract

**Purpose:**

This study develops a particle swarm optimization (PSO)-based assessment system for evaluating upper extremity and shoulder joint muscle strength with potential application to stroke rehabilitation. This study validates the system on healthy adult volunteers using surface electromyography and joint motion data.

**Methods:**

The system comprises a multimodal data acquisition module and a computational analysis pipeline. sEMG signals were collected non-invasively from the anterior, medial, and posterior deltoid muscles using bipolar electrode arrays. These signals are subjected to noise reduction and feature extraction. Simultaneously, triaxial kinematic data of the glenohumeral joint were obtained via an MPU6050 inertial measurement unit, processed through quaternion-based orientation estimation. Machine learning models, including Backpropagation Neural Network (BPNN), Support Vector Machines (SVM), and particle swarm optimization algorithms (PSO-BPNN, PSO-SVR), were applied for regression analysis. Model performance was evaluated using R-squared (*R* ^2^), Root Mean Square Error (RMSE), Mean Absolute Error (MAE), and Mean Bias Error (MBE).

**Results:**

The system successfully collected electromyographic and kinematic data. PSO-SVR achieved the best predictive performance (*R* ^2^ = 0.8600, RMSE = 0.3122, MAE = 0.2453, MBE = 0.0293), outperforming SVR, PSO-BPNN, and BPNN.

**Conclusion:**

The PSO-SVR model demonstrated the highest accuracy, which can better facilitate therapists in conducting muscle strength rehabilitation assessments.

**Significance:**

This system enhances quantitative assessment of muscle strength in stroke patients, providing a reliable tool for rehabilitation monitoring and personalized therapy adjustments.

## 1 Introduction

Stroke, a prevalent neurological disorder in China (Wu et al., 2019), is increasingly recognised for its significant risk of mortality and disability. Approximately 80% of acute stroke patients exhibit upper limb motor deficits, and up to 50%–60% of these individuals continue to experience sequelae after 6 months (Dawson et al., 2021). Despite a reduction in the mortality rate of stroke patients due to medical advancements, the incidence of stroke has risen alongside population growth and an ageing demographic (Ananth et al., 2023). The increasing prevalence of stroke has heightened the demand for rehabilitation services (Stinear et al., 2020). Neuroplasticity enables patients with upper limb dysfunction post-stroke to enhance upper limb function to a certain degree through rehabilitation within the initial 3 months (Xing and Bai, 2020; Hubbard et al., 2015; Dromerick et al., 2021). Early rehabilitation is crucial for the recovery of upper limb dysfunction post-stroke, aiding patients in optimising upper limb function and enhancing quality of life and independence (Cecchi et al., 2021). Enhancement of upper limb function has been identified as one of the clinically recognized research priorities for rehabilitation efforts (Rodgers et al., 2019). The evaluation of upper extremity functional impairment and the advancement of rehabilitation therapy must be conducted prior to the confirmation of a patient’s rehabilitation program. This indicates that in the comprehensive rehabilitation management of stroke, establishing a scientific and effective functional assessment system remains the essential component for enhancing patient prognosis quality (Liu et al., 2022).

Traditional clinical assessments for upper limb motor function post-stroke, such as the Fugl-Meyer Assessment (FMA) and Brunnstrom stages (Riahi et al., 2020; Hsieh et al., 2007; Feys et al., 1998; Cordes et al., 2024), offer standardized and repeatable evaluation across multiple domains. However, these tools rely heavily on manual scoring and clinical observation, leading to high subjectivity, limited quantification, and reduced efficiency. These limitations highlight the need for objective, data-driven assessment methods to support precise and individualized rehabilitation planning.

With advancements in technology, traditional medical methods are increasingly integrated with machine learning, artificial intelligence, and other interdisciplinary fields (Wei and Wu, 2023). Contemporary devices such as wearable sensors, upper limb rehabilitation robots, and virtual reality technology can be utilised for the assessment of upper limb motor function (Dewil et al., 2023; Chen X. et al., 2022; Tozlu et al., 2020). Prange and other scholars discovered that robotic-assisted therapy was more efficacious than traditional therapy for patients in post-stroke rehabilitation, particularly regarding the enhancement of motor control (Prange et al., 2009). Wearable sensors encompass Surface Electromyographic Signal (sEMG) sensors, inertial measurement units (IMUs), among others. Ruan et al. (2024) employed high-density sEMG signals to quantify muscle co-activation patterns in stroke patients, aiming to enhance the assessment of hand injuries in this population. Bishop et al. (2024) developed a wearable device using inertial sensors to quantify and characterize mobility and upper limb movements in stroke patients, an innovation with high accuracy, acceptability and usability. Wang et al. (2025) integrated sEMG signals to develop an upper limb rehabilitation assessment method utilising an enhanced dynamic time warping (DTW) algorithm. The enhanced DTW algorithm can more precisely evaluate the recovery of upper limb movement, encompassing range of motion, coordination, and accuracy.

For shoulder-specific assessment in upper limb rehabilitation, selecting appropriate movements is equally challenging (Agrebi et al., 2024). Demonstrated that utilizing an Specific Strength Device with throwing-specific movements significantly improved peak concentric and eccentric torque performance in nearly all major muscle groups assessed across both arms. The diagnostic validity of motor rehabilitation assessments is fundamentally contingent upon the recognition fidelity of movement pattern classification models. To address limitations in pattern discrimination accuracy inherent in conventional methodologies, this investigation implements a multimodal biosignal capture framework integrating a multi-channel bipolar sEMG acquisition array with an MPU6050-derived inertial measurement unit (IMU). This synergistic configuration enables synchronous recording of neuromuscular activation signatures (0–500 Hz bandwidth) and triaxial kinematic parameters during upper extremity functional movements, thereby establishing a robust data foundation for subsequent analytical processing. Subsequently, we utilize BPNN and support vector machine learning techniques to build a regression prediction model and integrate PSO-BPNN into the regression prediction model. Utilise SVM algorithms to construct a regression prediction model, employing root mean squared error (RMSE), coefficient of determination (R-Square, *R* ^2^), mean absolute error (MAE), and mean bias error (MBE) as evaluation metrics to assess the model’s performance and identify the algorithm with optimal overall efficacy for rehabilitation assessment.

## 2 Methods

**FIGURE 1:**
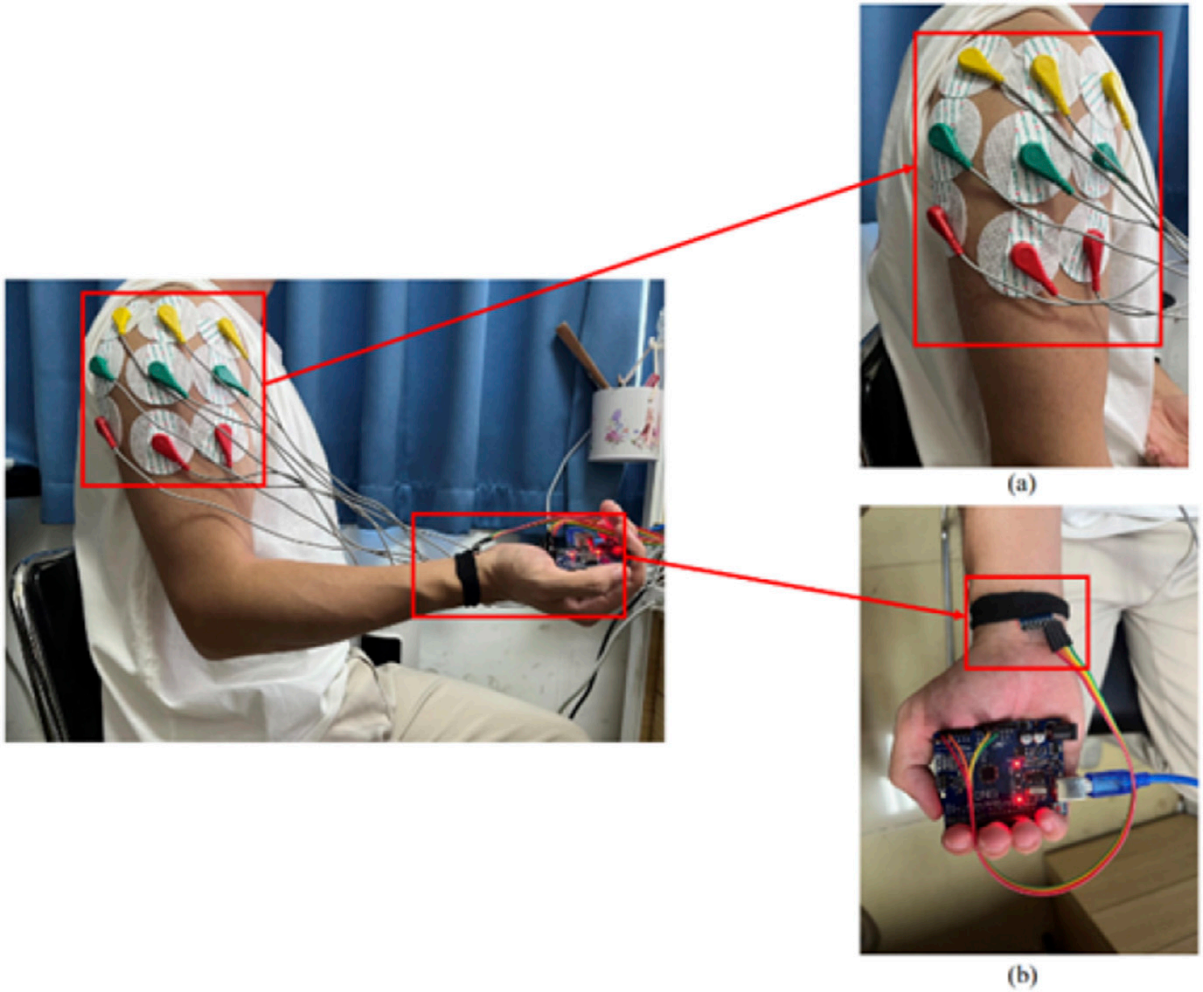
Placement of the upper extremity test device: **(a)** placed in the anterior, middle and posterior deltoid tracts **(b)** MPU6050 positioned on the distal limb.

**FIGURE 2:**
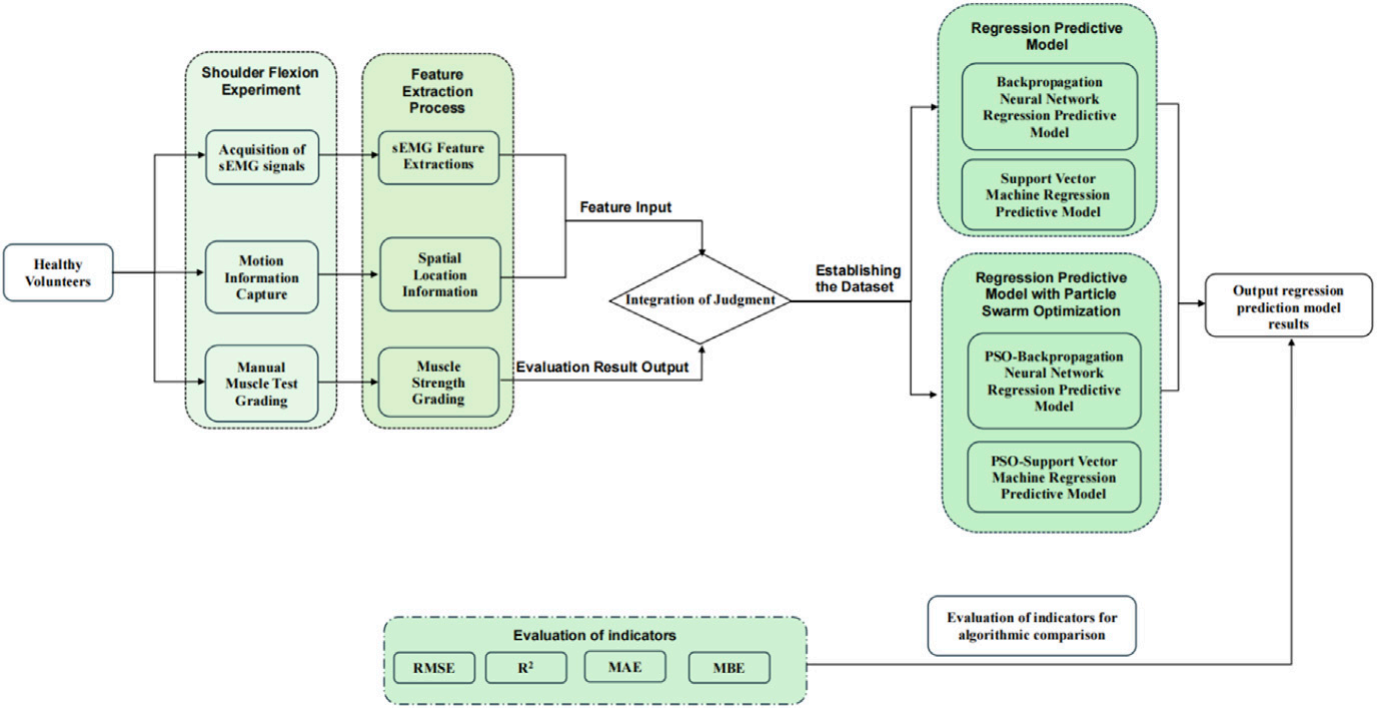
Research methods for predicting upper extremity muscle strength regression with sEMG and motion capture techniques.

The experimental protocol had asymptomatic adult volunteers perform a standardized anterior raise targeting the right glenohumeral joint. In these biomechanical tests, bipolar surface electrodes captured electromyographic activity in the three functional regions of the anterior deltoid, middle deltoid, and posterior deltoid, while an inertial motion capture system was used to monitor the triaxial trajectory of the ipsilateral limb. This bilateral sensor fusion strategy enabled comprehensive characterization of interlimb coordination patterns during unilateral load-bearing tasks. The sEMG sensor was utilised in conjunction with the MPU6050 inertial measurement unit during the experiment, as illustrated in Figure 1. Initially, data acquisition was conducted, followed by the extraction of kinematic feature parameters and the documentation of the subject’s muscle strength level utilising the unarmed muscle strength rating standard, which facilitated the construction of the machine learning regression model for predicting muscle strength levels. The predictive accuracy of the developed computational framework was rigorously evaluated through four statistical measures: R-Square (*R* ^2^) to assess goodness-of-fit, Root Mean Squared Error (RMSE) and Mean Absolute Error (MAE) to quantify error magnitude, along with Mean Bias Error (MBE) to detect directional errors. These evaluation metrics were strategically selected to provide comprehensive insights into model performance characteristics. The experimental methodology and analytical workflow are systematically presented through a schematic diagram in Figure 2, which outlines the sequential phases from data preprocessing to final validation.

### 2.1 sEMG signal acquisition and analysis

Muscles govern the movement of human limbs, and muscular strength measures the greatest force exerted by a muscle within specific constraints (Bohannon, 2019). Muscle force is typically not measurable directly; it must be assessed indirectly, utilizing methods such as EMG signal mapping and inverse kinematics (Borbély and Szolgay, 2017). EMG recordings fundamentally document bioelectrical patterns generated during musculoskeletal activation, serving as critical biomarkers for functional muscle evaluation and clinical assessment of neuromuscular health (Chen Z. et al., 2022). These non-invasive devices detect myoelectric signatures through electrode arrays positioned on the skin surface, measuring differential potentials across targeted muscle group (Wu et al., 2021). In rehabilitation science applications, sEMG technology demonstrates particular clinical value by enabling safe monitoring of motor unit recruitment patterns - as exemplified in Hsu et al. (2019) kinematic study analyzing post-stroke patients’ sit-to-stand transition mechanics through muscular activation sequences (Huang et al., 2020). Unlike invasive monitoring techniques, sEMG provides a safer, noninvasive method for recording muscle activity. It reduces clinical risk while preserving diagnostic reliability. This makes it suitable for various applications, such as tracking rehabilitation progress, assessing movement patterns, and analyzing muscle exertion.

#### 2.1.1 sEMG signal acquisition system

**FIGURE 3:**
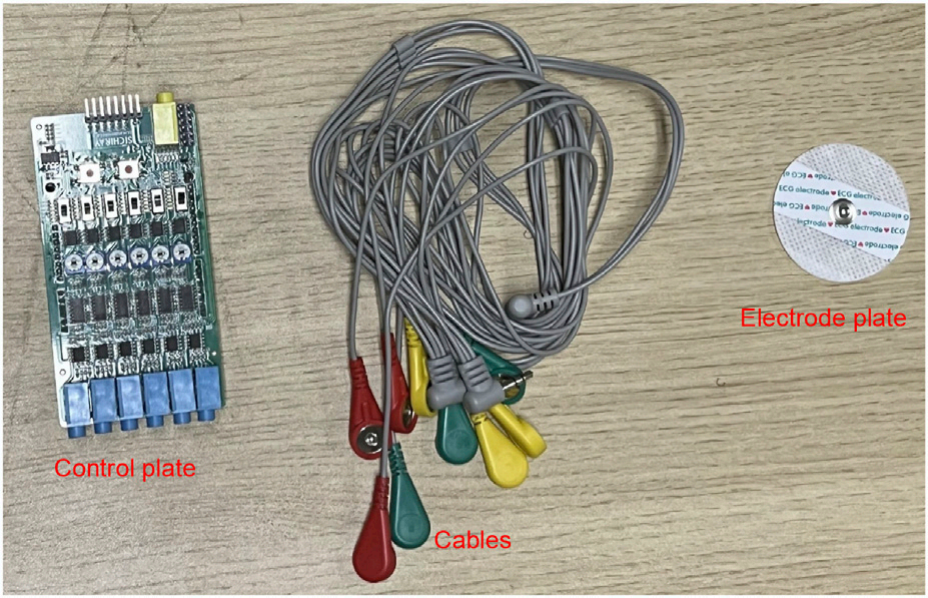
sEMG signal acquisition equipment.

A multi-channel sEMG acquisition system comprising six detection modules was implemented with a sampling rate of 10 kHz to record neuromuscular activity patterns, as detailed in the system schematic (Figure 3). sEMG signals were collected using a commercial multichannel system (Sichiray) with 6 surface electrodes. The system sampled at 10 kHz with a 16-bit resolution, analog input bandwidth of 20–450 Hz, and internal gain of 1,000 × . Input impedance exceeded 10 Megaohm, and built-in analog filters were used to reduce motion artifacts. The sEMG signals were sampled at 10 kHz to capture the full bandwidth of muscle activity, which typically ranges between 10 Hz and 500 Hz. According to the Nyquist sampling theorem, a minimum sampling rate of 1–2 kHz is sufficient to avoid aliasing for typical sEMG signals (Baraniuk et al., 2017); however, we selected 10 kHz to enable high time-resolution analysis and preserve signal fidelity during preprocessing and feature extraction, especially for transient muscle bursts. The inertial measurement unit (MPU6050) was sampled at 100 Hz, which is sufficient for capturing upper-limb joint kinematics during voluntary motion, as human joint dynamics rarely exceed 10–15 Hz. sEMG and IMU data were temporally aligned via timestamp matching using a shared microcontroller clock, ensuring synchronized multimodal data acquisition. This investigation focused on upper extremity biomechanical responses through systematic analysis of three distinct segments within the deltoid muscle complex - specifically the clavicular, acromial, and spinal fascicles.

The electrode configuration was standardized according to the schematic shown in Figure 1a, featuring color-coded placement protocols. The reference electrode (denoted in red) maintained consistent alignment along the myofascial boundary, while the detection electrodes (chromatically differentiated as yellow and blue) were positioned over the motor point regions of target muscles. These non-invasive surface electrodes were specifically assigned for bipolar signal detection in sEMG monitoring applications.

The experimental protocol was designed to quantify neuromuscular activation characteristics across the three deltoid subdivisions during dynamic shoulder flexion movements. Through this methodological framework, temporal-spatial activation patterns were systematically analyzed to elucidate the synergistic coordination mechanisms underlying humeral elevation in the sagittal plane.

#### 2.1.2 Signal preprocessing and feature extraction

**FIGURE 4:**
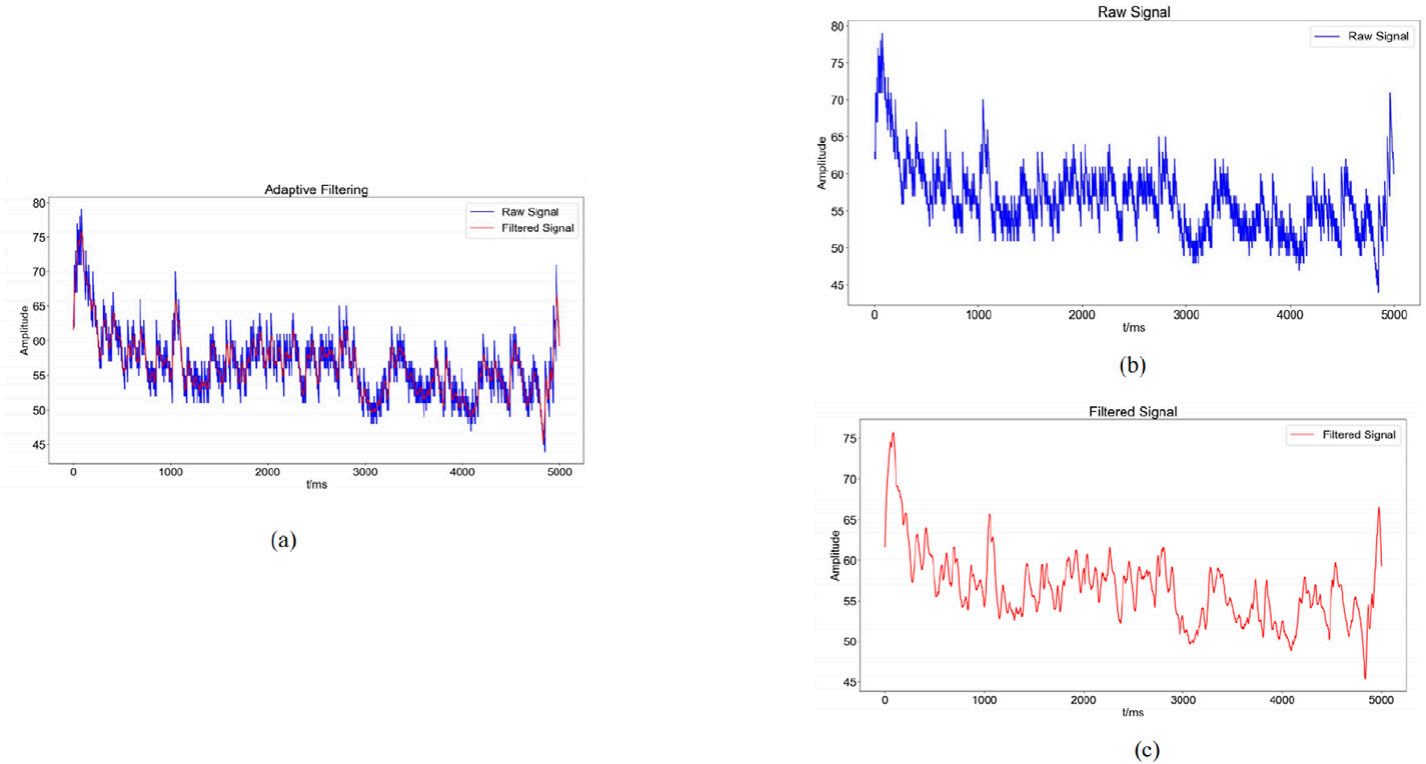
Graphs of noise reduction results (**(a)**: Comparison between the raw sEMG signal (blue) and the denoised sEMG signal (red) obtained through adaptive filtering; **(b)**: blue waveform indicates the change of the original sEMG signal; **(c)**: red waveform indicates the sEMG signal after noise reduction by adaptive filtering).

The sEMG signal reflects important information about the activity of subcutaneous muscle tissue. However, because sEMG is very weak and therefore susceptible to various types of noise, the use of sEMG usually requires the use of specific filters for noise reduction of sEMG signals (Guo et al., 2021). In this experiment, a Savitzky-Golay filter was used for noise reduction. This filter has satisfactory denoising performance while preserving the signal trend and width of the sEMG. Its advantages include the preservation of characteristic signal information and superior noise resistance. In this experiment, the Savitzky-Golay filter is employed to attenuate the noise of the original sEMG signal. The key parameters governing the filter’s operation are window length and polynomial order (polyorder). In this study, the window length is set to 51, meaning that each smoothing computation considers 51 data points, or equivalently, 51 time units. This parameter influences the filter’s ability to suppress noise, as a fixed-length window may not always effectively reduce signal contamination. The polyorder, defined as the polynomial degree used for fitting data within each window, is assigned a value of 3 in this experiment. This indicates that cubic polynomials are utilized to approximate the data trend in each segment. The window length of 51 and polynomial order of 3 were selected for the Savitzky-Golay filter based on a balance between noise suppression and preservation of signal morphology. A longer window helps smooth high-frequency noise, while a cubic polynomial maintains the essential features of sEMG waveforms such as peak structure and slope continuity. Preliminary tests comparing this configuration with moving average and Butterworth low-pass filters showed that the Savitzky-Golay filter achieved superior denoising while preserving temporal characteristics of the muscle activation signal. Therefore, it was selected as the optimal preprocessing method for our study. The results of this filtering process are presented in the accompanying Figure 4. We refer to the literature for methods that are consistent with biomechanical signal acquisition (Dhahbi et al., 2017). Prior to sEMG data acquisition, the skin at electrode placement sites was prepared by shaving excess hair, lightly abrading the surface with fine sandpaper, and cleaning with 70% alcohol to reduce impedance and motion artifacts. In line with standard sEMG acquisition guidelines. Signal quality was assessed in real time by visually inspecting baseline noise and ensuring stable waveform morphology during low-force contractions. Channels with unstable baselines, excessive motion artifact, or powerline interference were excluded or reconfigured before data collection.

This work used three time-domain features—Root Mean Square (RMS), integrated EMG (iEMG), and Mean Absolute Value (MAV)—along with the Median Frequency (MF) as a frequency-domain feature to assess the collected sEMG signals. These features capture signal energy, recruitment efficiency, and fatigue state, aligning closely with the physiological characteristics of muscle strength impairment and recovery trajectory in post-stroke rehabilitation.

Equation 1 of sEMG signals represents the root mean square value of all amplitudes within a specified time interval, illustrating the average variation characteristics of sEMG over time, indicating the energy value of sEMG generated during muscular activities, and serving to assess the contribution of specific muscles in executing various movement processes.

$$RMS=\sqrt{\frac{1}{N}\overset{N}{\underset{i=1}{\sum }}x^{2}\left(i\right)}$$ (1)

Equation 2 of the sEMG signal represents the cumulative area under the curve per unit time following the rectification and smoothing of the recorded sEMG signal. This metric indicates the total discharge of motor units over a specified duration, reflecting the temporal variations in the intensity of the sEMG signal.

$$iEMG=\frac{1}{N}\overset{t+T}{\underset{t=1}{\sum }}\left|EMG\left(t\right)\right|dt$$ (2)

Where: EMG(t) represents the acquired electromyography signal, where t denotes the time variable, and T corresponds to the signal’s period.

The Equation 3 represents the average of the absolute values of the amplitude of the sEMG signal during a certain time interval, typically employed to evaluate muscle contraction strength and tiredness.

$$MAV=\frac{1}{N}\overset{N}{\underset{i=1}{\sum }}\left|x\left(i\right)\right|$$ (3)

Here, N represents the total number of acquired surface EMG signal data points, and x(i) is the ith data point in the signal sequence, which is often utilized to ascertain the intensity amplitude of the motion signal.

Equation 4 is the central value of discharge frequency during muscle contraction, commonly utilized to evaluate muscle contraction strength and exhaustion, typically diminishing as exercise duration increases.

$$MF=\frac{1}{2}\int_{0}^{\infty }PSD\left(f\right)df$$ (4)

where PSD represents the power distribution of the sEMG signal across different frequencies, while df refers to the sampling frequency.

**FIGURE 5:**
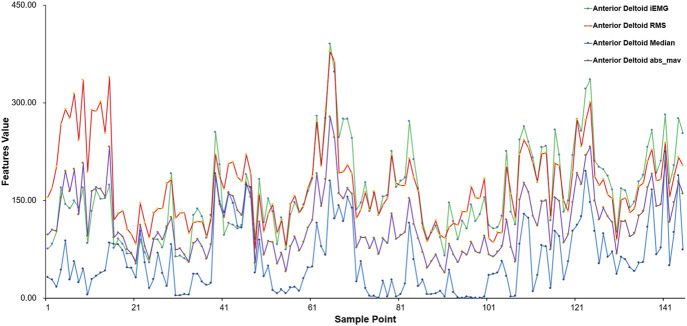
Results of feature extraction for different sample points (anterior deltoid fascicle).

This includes sample points from various subjects performing different movements, using the anterior deltoid fasciculus as a case study. Figure 5 presents the eigenvalue distribution for each sample point, highlighting notable variations in eigenvalues among different movements. The eigenvalues of sEMG signals vary according to the physical condition of each subject, ensuring the integrity of the original data.

### 2.2 Inertial sensor acquisition and analysis

During upper limb rehabilitation after a stroke, shoulder joint movement often results from compensatory actions of the scapula or trunk. Minimizing these compensatory movements can improve rehabilitation effectiveness, which can be achieved through biofeedback mechanisms, such as inertial sensing units.

An inertial measurement unit (IMU) is a wearable sensor system that integrates gyroscopes, accelerometers, and magnetometers. Inertial measurement units have a wide range of applications either in limb movement capture or recognition (Meng et al., 2023). Columns such as (Mahmoud et al., 2021) used inertial sensors, Kinect camera and sEMG sensors in combination with occupational therapy (OT) to assess upper limb function in stroke patients. Passon et al. (2020) utilized wearable IMUs alongside an end-effector robot to achieve precise motion tracking during rehabilitation and assess the effectiveness of feedback-based motor assistance. Kim et al. (2020) employed IMU-equipped sensors to collect elbow motion data, using machine learning algorithms such as random forests to analyze spasticity. Acharya et al. (2022) applied MPU6050 inertial sensors to measure upper limb movement direction and analyzed it against hand trajectories recorded by Kinect sensors.

#### 2.2.1 MPU6050 acquisition device

The MPU6050 is a six-degree-of-freedom inertial sensor (Tjhai and O’Keefe, 2019). The sensor module consists of the MPU6050 and an Arduino UNO, which analyzes the angle of motion and acceleration using the direction calculation method implemented in the Arduino IDE. In this study, the position of the MPU6050 is shown in Figure 1b. The MPU6050 sensor was affixed using double-sided medical adhesive tape to the distal third of the lateral aspect of the right upper arm, approximately 5 cm proximal to the lateral epicondyle of the humerus, avoiding areas with excessive soft tissue or bony protrusions. The sensor was aligned longitudinally with the humeral shaft, with its X-axis facing anteriorly. Elastic bands were used to further secure the sensor and minimize motion artifacts during shoulder flexion. This placement ensured consistent angular measurements of the glenohumeral joint across participants.

#### 2.2.2 MPU6050 acquisition and feature processing

**FIGURE 6:**
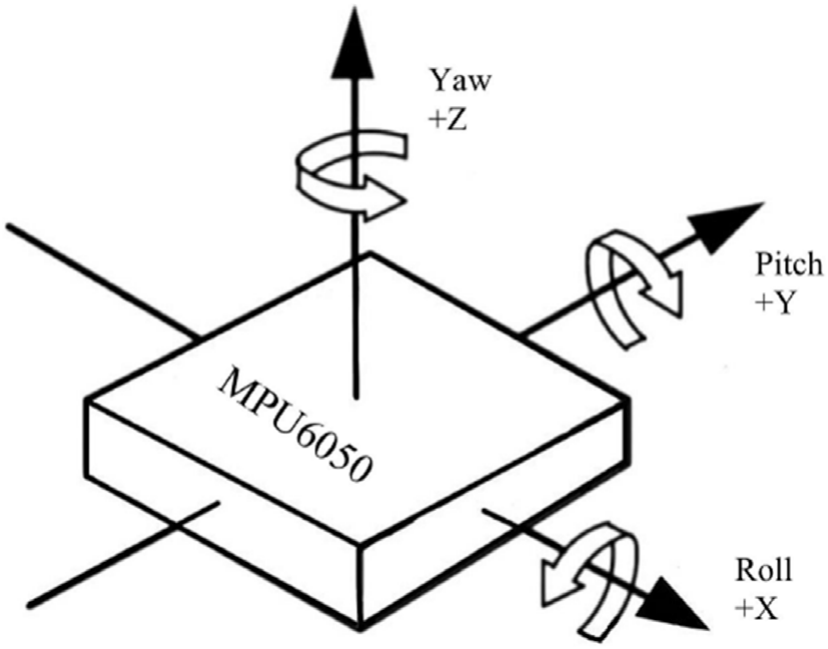
MPU6050 three-axis offset angle.

During the experiment, the MPU6050 sensor was placed at the distal end of the upper limb and the shoulder position was fixed to infer the shoulder joint motion angle. In this paper, the three-axis acceleration, three-axis angular velocity, and three-axis bias angle acquired by the MPU6050 are used to analyze the data, in which the rotation around the Z-axis is the heading angle (yaw), the rotation around the Y-axis is the pitch angle (pitch), and the rotation around the X-axis is the traversing roll angle (roll) (Figure 6). The MPU6050 has a three-channel gyroscope and a three-channel accelerometer internally, and thus the MPU sensors are able to output the three-axis acceleration (Acc), and three-axis angular velocity (Gyro).

The gyroscope combines acceleration and angular velocity over a time interval to determine Equation 5, as expressed in the following formula:

$$\left\{\begin{array}{c} \text{roll}=K\cdot \left({\text{roll}}_{\text{gyro}}+\omega_{x}\cdot \Delta t\right)+\left(1-K\right)\cdot {\text{roll}}_{\text{acc}}\\ \text{pitch}=K\cdot \left({\text{pitch}}_{\text{gyro}}+\omega_{y}\cdot \Delta t\right)+\left(1-K\right)\cdot {\text{pitch}}_{\text{acc}}\\ \text{yaw}={\text{yaw}}_{\text{gyro}}+\omega_{z}\cdot \Delta t \end{array}\right.$$ (5)

The fusion coefficient K was empirically set to 0.4 based on repeated experiments conducted during system testing. This value provided a practical balance between responsiveness and noise suppression for shoulder joint motion tracking. Although fixed in this study, K could be adaptively tuned in future implementations depending on motion intensity or application scenarios.

### 2.3 Analysis methods for predictive modeling

As machine learning continues to evolve, the integration of medicine and engineering continues to advance, especially in the diagnostic process and in the monitoring and categorization of neurological activity (Wang S. et al., 2018; Górriz et al., 2019; Gaur et al., 2019; Zhang et al., 2018). Modern techniques, such as Random Forests and Support Vector Machines, offer superior performance over traditional methods in various applications (Yu et al., 2018; Zhang et al., 2019). When applied to muscle data, these algorithms have proven effective in classifying movements and detecting irregularities in muscle function (Gómez-Vilda et al., 2019; Burns et al., 2020; Dai and Hu, 2019). This study employs sEMG and movement signals, utilizing a Backpropagation (BP) neural network and a Support Vector Machine (SVM) to predict muscle regression during motion and evaluate muscle strength recovery. Existing studies have shown (Wang S. et al., 2024) that initial progress has been made in machine learning-based assessment of rehabilitation systems, but limitations in model optimization remain. The BP neural network is prone to local optima and is sensitive to initial weights and biases; when addressing complex issues, the support vector machine may encounter overfitting or underfitting. To mitigate these challenges, this study incorporates the PSO algorithm into both the BPNN and the support vector machine regression algorithm, enhancing algorithm performance and improving the accuracy and stability of the regression prediction model.

The particle swarm algorithm, commonly referred to as the bird flock foraging algorithm, is a stochastic search method grounded in collective cooperation, inspired by the foraging behavior of avian flocks (Shu et al., 2023). It has been extensively utilized in unconstrained, constrained, and other defined problems because to its straightforward implementation, rapid convergence, and little parameter adjustment requirements (Cui et al., 2022; Liu et al., 2020; Cao et al., 2020). Equation 6 is an iterative method to find the optimal solution from random solutions and its algorithm is formulated as follows:

$$v_{i+1}=\omega v_{i}+c_{1}{\text{rand}}_{1}\left({\text{pbest}}_{i}-x_{i}\right)+c_{2}{\text{rand}}_{2}\left({\text{gbest}}_{i}-x_{i}\right)$$ (6)

Rand represents a random number within the interval (0, 1), while $c_{1}$ and $c_{2}$ are learning coefficients. $c_{1}$ denotes the influence of the particle’s own experiences on its subsequent actions, specifically the acceleration weight directing the particle towards its individual optimal position, pbest. Conversely, $c_{2}$ reflects the influence of the experiences of other particles, serving as the acceleration weight guiding the particle towards the group optimal position, gbest. The parameter ω signifies the inertia factor; a larger ω enhances the particle’s global optimization capabilities while diminishing its local optimization abilities, whereas a smaller ω weakens global optimization capabilities while enhancing local optimization abilities. The key hyperparameters in SVR (penalty parameter C and kernel coefficient γ ) and in PSO (cognitive coefficient $c_{1}$ , social coefficient $c_{2}$ , and inertia weight w ) were determined empirically. Specifically, we conducted manual tuning within practical ranges informed by prior studies and commonly used settings: C∈[1,100] , γ∈[0.001,1] , $c_{1},c_{2}\in \left[0.5,2.5\right]$ , and w∈[0.4,0.9] . The final values were selected based on repeated testing that yielded the best performance (highest $R^{2}$ , lowest RMSE) on the training data. While not exhaustive, this empirical tuning approach balances computational cost and model accuracy.

After extracting a total of 21 features from sEMG and IMU signals, all features were retained for model training. This choice was based on preliminary tests showing minimal multicollinearity and no significant performance gain from standard dimensionality reduction techniques. Nevertheless, we acknowledge that feature selection methods could be explored in future work to optimize model efficiency and interpretability.

#### 2.3.1 BP neural network regression prediction models

BPNN is a multilayer feedforward network trained according to the error backpropagation algorithm. It is currently widely used in the medical field (Yan et al., 2022; Wu et al., 2024; Han, 2024). The BPNN comprises an input layer, a hidden layer, and an output layer, with the interlayer weights determined by signal forward propagation and error backpropagation to construct the BPNN.

#### 2.3.2 Support vector machine regression prediction models

Support Vector Machines (SVM) is a supervised learning approach commonly used for both classification and regression tasks, where a subset of the available data is employed for model training. In regression analysis, this methodology is specifically known as Support Vector Regression (SVR). SVR is particularly effective in handling nonlinear relationships in high-dimensional spaces, showcasing strong performance in complex scenarios. Exhibit strong regression performance and are extensively utilized in clinical, physical, chemical, and engineering domains (Gao and Liu, 2024; Wu et al., 2022; Lin et al., 2021; Sahoo et al., 2022). In the regression problem, Support Vector Regression (SVR) establishes a “margin” on either side of the linear function, permitting a deviation of ϵ , and refrains from calculating the loss for all samples within this margin. The optimized model is obtained by minimizing the overall loss function while simultaneously maximizing the margin. To allow for flexibility in the margin, slack variables, denoted as ξ and $\xi^{*}$ , are introduced. These variables enable a controlled degree of relaxation on both sides of the margin and are used to measure the errors in the model’s predictions is utilized to quantify the magnitude of error in the model’s projected output, and the loss function is Equation 7:

$$\left\{\begin{array}{c} \underset{w,b,\xi_{i},\xi_{i}^{*}}{min}\frac{1}{2}{\left\|w\right\|}^{2}+C\overset{m}{\underset{i=1}{\sum }}\left(\xi_{i},\xi_{i}^{*}\right)\\ s.t.\;\;f\left(x_{i}\right)-y_{i}\leq \varepsilon +\xi_{i}\\ y_{i}-f\left(x_{i}\right)\leq \varepsilon +\xi_{i}\\ \xi_{i}\geq 0,\xi_{i}^{*}\geq 0,\;i=1,2,\ldots ,m \end{array}\right.$$ (7)

#### 2.3.3 PSO-BPNN regression prediction model

In the PSO-BPNN, each particle signifies a weight configuration of the neural network, while the particle’s velocity denotes the rate of weight alteration. The optimal weight configuration is determined by updating the particles’ velocity and position via the PSO algorithm (Wang Y. et al., 2024). The BPNN is utilized for training and optimizing network weights and thresholds to reduce output error. The PSO-BP neural network merges the global search abilities of PSO with the local search abilities of the BP neural network. This combination allows for efficient and quick solutions to complex nonlinear problems.

#### 2.3.4 PSO-SVM regression prediction modeling

PSO-SVM integrates the PSO technique with the SVM algorithm to enhance the parameters and model of SVM. PSO is employed to optimize parameters in SVM, including kernel function parameters and the penalty parameter, to enhance the performance and generalization of SVM (Zhao et al., 2024). In PSO-SVM, each particle signifies a potential solution (a set of parameters) for SVM, the particle’s position denotes the parameter values, and the particle’s velocity is employed to modify these parameter values. The particle modifies its position and velocity based on the assessment outcomes of the objective function to identify the ideal parameter combination, hence enhancing the performance of the SVM.

#### 2.3.5 Assessment of indicators

To assess the efficacy of comparing those machine learning models, specific evaluation metrics are typically employed to indicate the accuracy of the predictive model. Four metrics were used in this study to determine the best regression method among the four algorithms. Equation 8, was used to measure the accuracy of machine learning predictions (Gao and Liu, 2024). The Equation 9 is the square root of the mean of the squared differences between the actual values and the predicted values. The lower the RMSE, the higher the predictive accuracy of the model. Equation 10 measures the average of the absolute differences between the actual values and the predicted values. The lower the MAE, the more accurate the model’s predictions. Equation 11 assesses the direction of error in a model by calculating the average difference between predicted and actual values. A positive MBE indicates that the predicted values are higher than the actual values, while a negative MBE indicates the opposite, and MBE close to zero indicates that the predictive model has minimal bias.

$$R^{2}=1-\frac{\sum_{i=1}^{n}{\left(Actual\;value_{i}-Predicted\;value_{i}\right)}^{2}}{\sum_{i=1}^{n}{\left(Actual\;value_{i}-Average\;of\;outputs\right)}^{2}}$$ (8)

$$RMSE=\sqrt{\frac{\sum_{i=1}^{n}{\left(Actual\;value_{i}-Predicted\;value_{i}\right)}^{2}}{n}}$$ (9)

$$MAE=\frac{1}{n}\overset{n}{\underset{i=1}{\sum }}\left|Actual\;value_{i}-Predicted\;value_{i}\right|$$ (10)

$$MBE=\frac{1}{n}\overset{n}{\underset{i=1}{\sum }}Actual\;value_{i}-Predicted\;value_{i}$$ (11)

This study evaluates the performance of machine learning regression models by comparing four assessment metrics: *R* ^2^;, RMSE, MAE, and MBE. All regression algorithms are implemented using Python. To ensure that the observed performance differences among the four regression models were not due to chance, we performed repeated-measures ANOVA on the cross-validated performance metrics (*R* ^2^;, RMSE, MAE, MBE), followed by Bonferroni correction for multiple comparisons. This correction reduces the risk of Type I error associated with evaluating multiple models simultaneously.

## 3 Experimental design and analysis

### 3.1 Experimental protocol design and data acquisition

This study recruited fifteen healthy volunteers. All participants were medically cleared, with no history of limb injuries, consciousness disorders, or other relevant medical conditions, and reported no substance abuse. The study protocol was approved by the Institutional Review Board of Guangdong Provincial Hospital of Traditional Chinese Medicine (Approval No. YE 2024-245-01). In this study, a six - channel EMG muscle electricity sensor collected sEMG signals, and an MPU6050 inertial sensor gathered motion data. Considering human physiological structures, for each participant, the system simultaneously collected sEMG signals from three parts of the right upper limb deltoid: anterior deltoid, middle deltoid, posterior deltoid.

In order to exclude the influence of skin surface magazines such as sweat, the subjects cleaned the skin around the tested muscles with 75% alcohol before the examination. Subjects sat in a seated position with the elbow joint fully extended during the shoulder flexion exercise. The right upper limb was lifted from the trunk lateral midline and returned to the trunk lateral midline for a complete shoulder flexion exercise. Each participant completed 10 shoulder flexion exercises at different angles. With the help of a professional rehabilitation therapist, they also underwent unarmed muscle strength tests based on the MMT grading scale (detailed in Table 1). A total of 150 data points were collected from 15 subjects. However, due to muscle fatigue, only 145 data points were usable. Muscle strength was graded 0–5 on the MMT scale, with grades 2–5 selected for examination and discussion. Participants with MMT grades of 0 or 1 were excluded from the study because individuals with such low muscle strength are typically unable to produce sufficient voluntary movement or detectable sEMG signals. Including these grades would result in unreliable data and inconsistent muscle activation profiles, making it difficult to train robust models. Therefore, only participants with MMT grades 2 through 5 were included to ensure signal quality and task feasibility.

**TABLE 1:** MMT muscle strength grading criteria.

Rank	Manifestations
0	No muscle contraction whatsoever
1	Muscle contractions are palpable, but there is no joint movement
2	Full joint range of motion in a gravity-defying position
3	Full joint range of motion against gravity, but not against resistance
4	Able to move against gravity and some resistance
5	Motion that resists gravity and full resistance

Given the relatively small dataset (n = 15), we adopted measures to mitigate overfitting. These included five-fold cross-validation during model training and performance evaluation, and performance consistency checks across folds. Moreover, we prioritized relatively simple model architectures and avoided excessive parameter tuning. Nonetheless, future work with larger and more diverse populations is planned to enhance generalizability.

### 3.2 Analysis of experimental results

The experiment constructed a dataset from the collected sEMG and exercise data, extracting 21 features from the iEMG signals of the anterior, middle, and posterior regions of the deltoid muscle fascia, including RMS, MAV, MF, Gyrox, Gyroy, Gyroz, Accx, Accy, Accz, Pitch, Roll, and Yaw obtained from the MPU6050. The rehabilitation therapist used the MMT scale to quantify the magnitude of muscle strength during exercise, and the resulting muscle strength was used as the output. A dataset was formed with characteristics as the independent variable and muscle force output as the dependent variable, and standardization was done using a linear transformation function Equation 12 to prevent different variables from being affected by different units. The dataset was then divided into a training set and a test set in 7:3, and the test set was tested using the model in the training set. Finally, four evaluation metrics - *R* ^2^, RMSE, MAE and MBE - were used to assess the performance of the model (Khan et al., 2021).

$$y=\frac{x-Min_{x}}{Max_{x}-Min_{x}}$$ (12)

#### 3.2.1 Results of the BP neural network regression model

**FIGURE 7:**
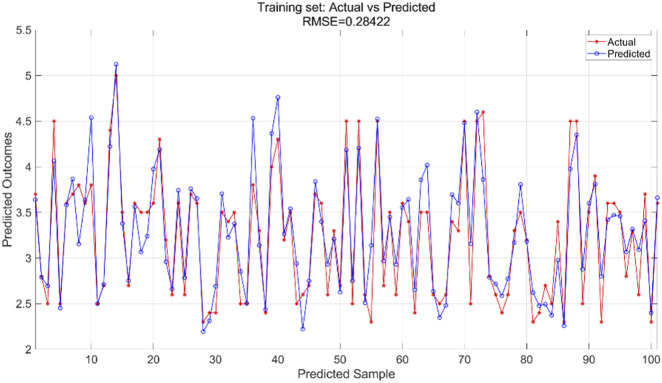
Training set training predicted and actual value results.

**FIGURE 8:**
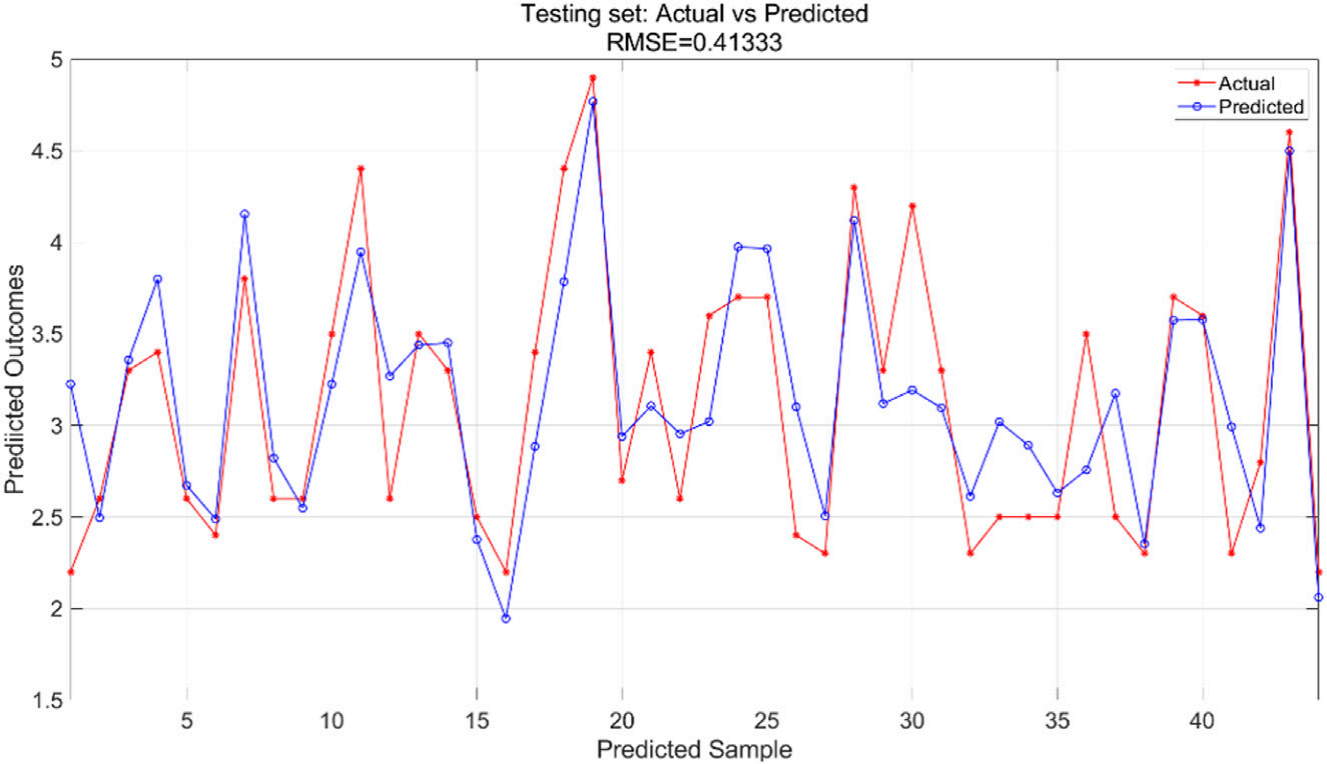
Testing set prediction model predicted and actual value results.

We used a BP neural network regression model with data for all variables. We adjusted the number of neurons in the hidden_layer_sizes and the number of max_iter. The number of iterations indicates the maximum number of iterations allowed during training. In this experiment, we configured the model to contain a hidden layer of 5 neurons and set the total number of iterations to 1,000. We train the BP neural network regression model using the training set and then apply it to the test set for prediction. The training results for the training set are shown in Figure 7 and the prediction results for the test set are shown in Figure 8.

The BPNN regression model was trained using the training dataset, which helped the model learn the underlying patterns and relationships in the data. Figure 7 presents a comparison of the predicted versus actual values within this training dataset, showing an RMSE of 0.28422, indicating a relatively good fit of the model to the training data. For the test dataset, which was not used during the training phase, the trained model was applied to make predictions. Figure 8 illustrates the comparison between the predicted and actual values in the test dataset, resulting in an RMSE of 0.41333. This higher RMSE value suggests that while the model performed well on the training data, there is some degree of generalization error when applying the model to unseen data, which is typical in machine learning tasks.

#### 3.2.2 Results of support vector machine regression modeling

**FIGURE 9:**
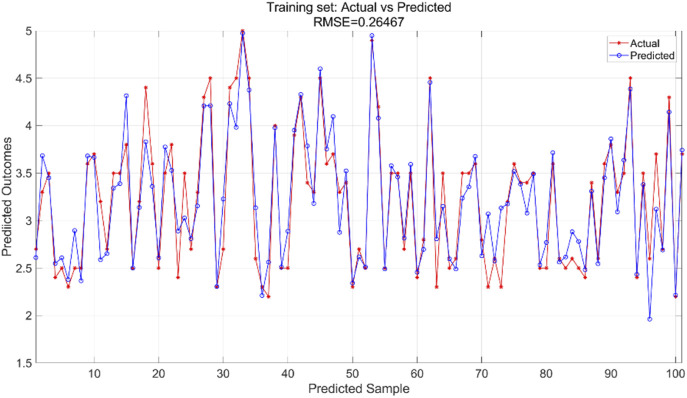
Training set training predicted and actual value results.

**FIGURE 10:**
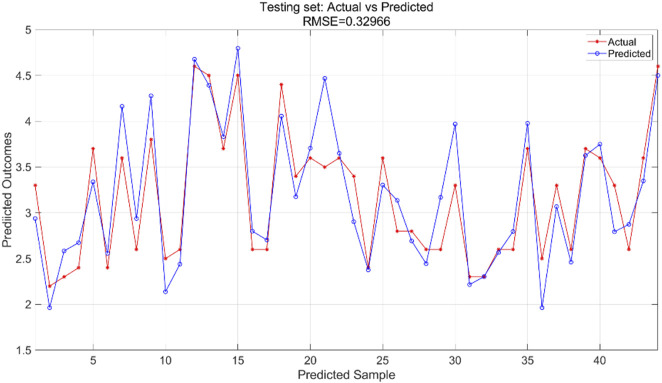
Testing set prediction model predicted and actual value results.

All variables are incorporated into the SVR model, which is configured with essential parameters: kernel function and associated parameters, regularization parameter (C), and error tolerance parameter (epsilon). The kernel function transforms input features into a high-dimensional space, enabling linear separation of nonlinear relationships. The regularization parameter regulates model complexity and fault tolerance, while the error tolerance parameter sets the allowable margin of error during model fitting. In this experiment, we utilized an RBF kernel with gamma = 0.1, regularization parameter C = 10, and error tolerance parameter = 0.1. The support vector machine regression model is trained using the training and test sets to predict outcomes, yielding training results for the training set (Figure 9) and prediction results for the testing set (Figure 10).

The SVR model was trained using the training dataset, which enabled the model to learn underlying patterns in the data. Figure 9 presents a comparison between the model’s predictions and the actual values within the training set, achieving an RMSE of 0.26467, indicating a good fit to the training data. For the test dataset, which was not seen during the training phase, the pre-trained model was applied to make predictions. Figure 10 displays the comparison between the predicted and actual values in the test set, resulting in an RMSE of 0.32966. This value, while higher than the training RMSE, suggests that the model maintains reasonable predictive performance on unseen data, with the difference between training and test performance being expected in supervised learning tasks.

#### 3.2.3 Results of PSO-BP neural network regression modeling

**FIGURE 11:**
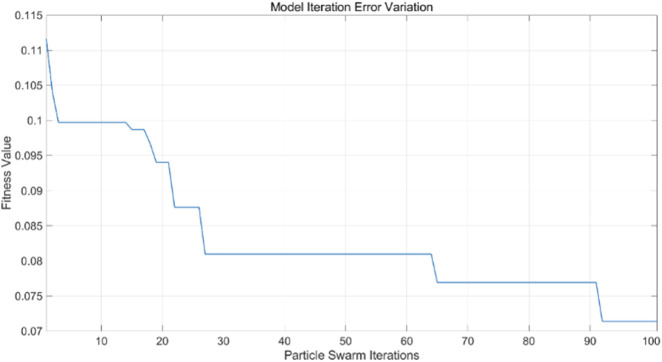
Iteration of PSO-BP model error curve.

**FIGURE 12:**
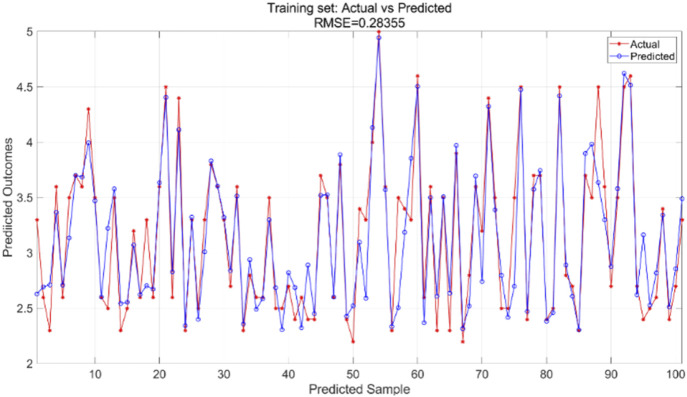
Training set training predicted and actual value results.

**FIGURE 13:**
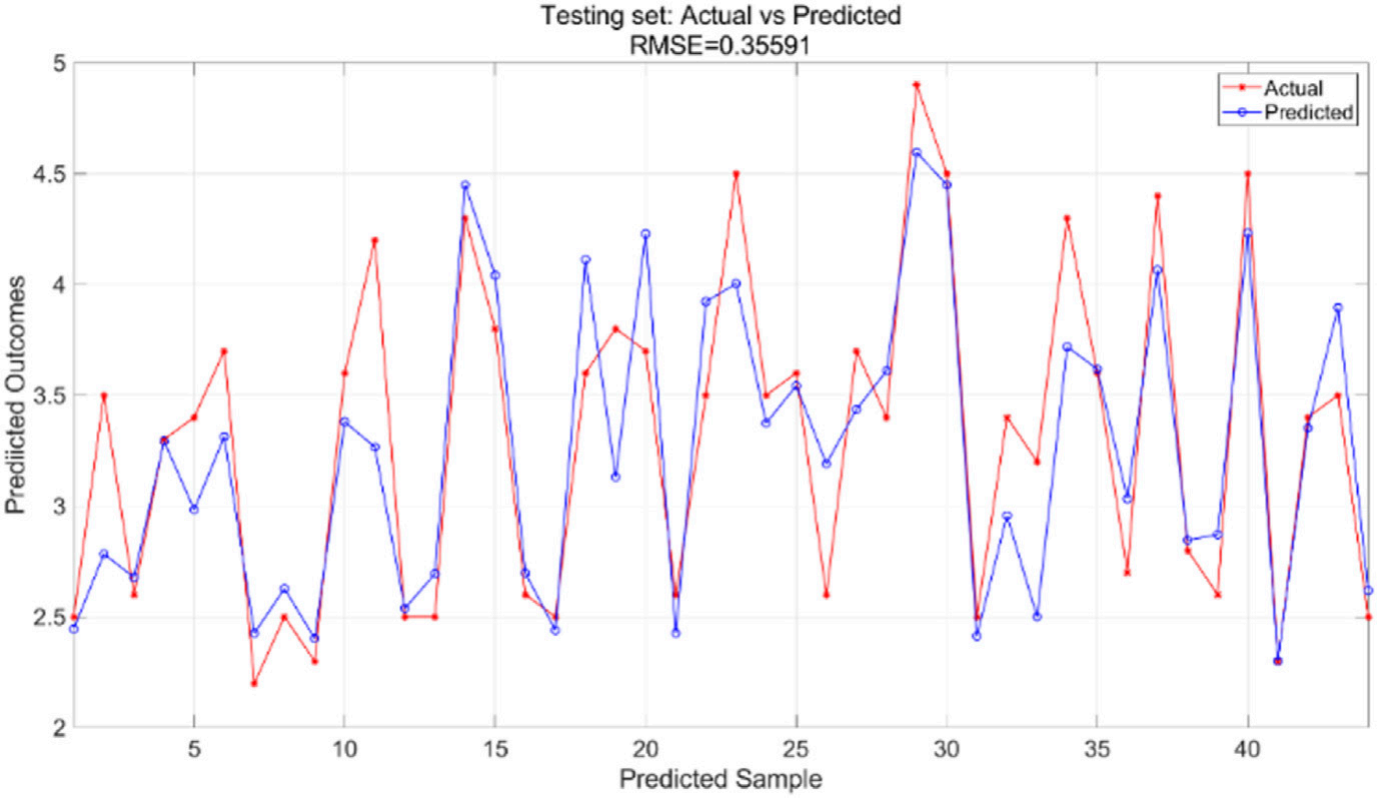
Testing set prediction model predicted and actual value results.

All variable data are incorporated into the PSO-BP neural network regression model, with model parameters modified according to particle swarm optimization requirements. This includes adjustments to the learning factors of the particle swarm, the number of iterations, and the size of the particle swarm population, based on the BP neural network. In this experiment, the learning factors $c_{1}$ and $c_{2}$ are set at 4.494, the number of particle swarm iterations is 100, and the particle swarm population size is 5. The findings of the PSO-BP neural network regression model are compared with the actual values from both the training and test sets. The training and test sets were utilized to train the PSO-BP neural network regression model for result prediction, yielding iteration error results (Figure 11), training set outcomes (Figure 12), and testing set predictions (Figure 13).

The particle swarm optimization algorithm delineates the error curve relative to the number of iterations. Figure 11 demonstrates that with 100 iterations, the overall model error ranges from 0.07 to 0.075. The training set primarily facilitates the training of the PSO-BP neural network regression model. Figure 12 illustrates the comparative results between predicted and actual values in the training set, yielding an RMSE value of 0.28355. The testing set is evaluated using the established model. The trained model predicts the test set, as illustrated in Figure 13, which compares the predicted and actual values; the RMSE is 0.35591.

#### 3.2.4 PSO-SVM regression model results

**FIGURE 14:**
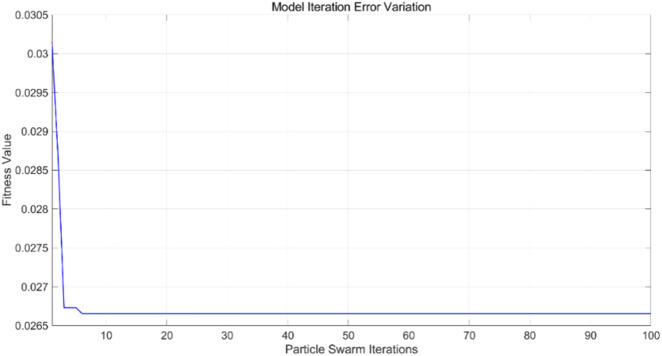
Iteration of PSO-SVR model error curve.

**FIGURE 15:**
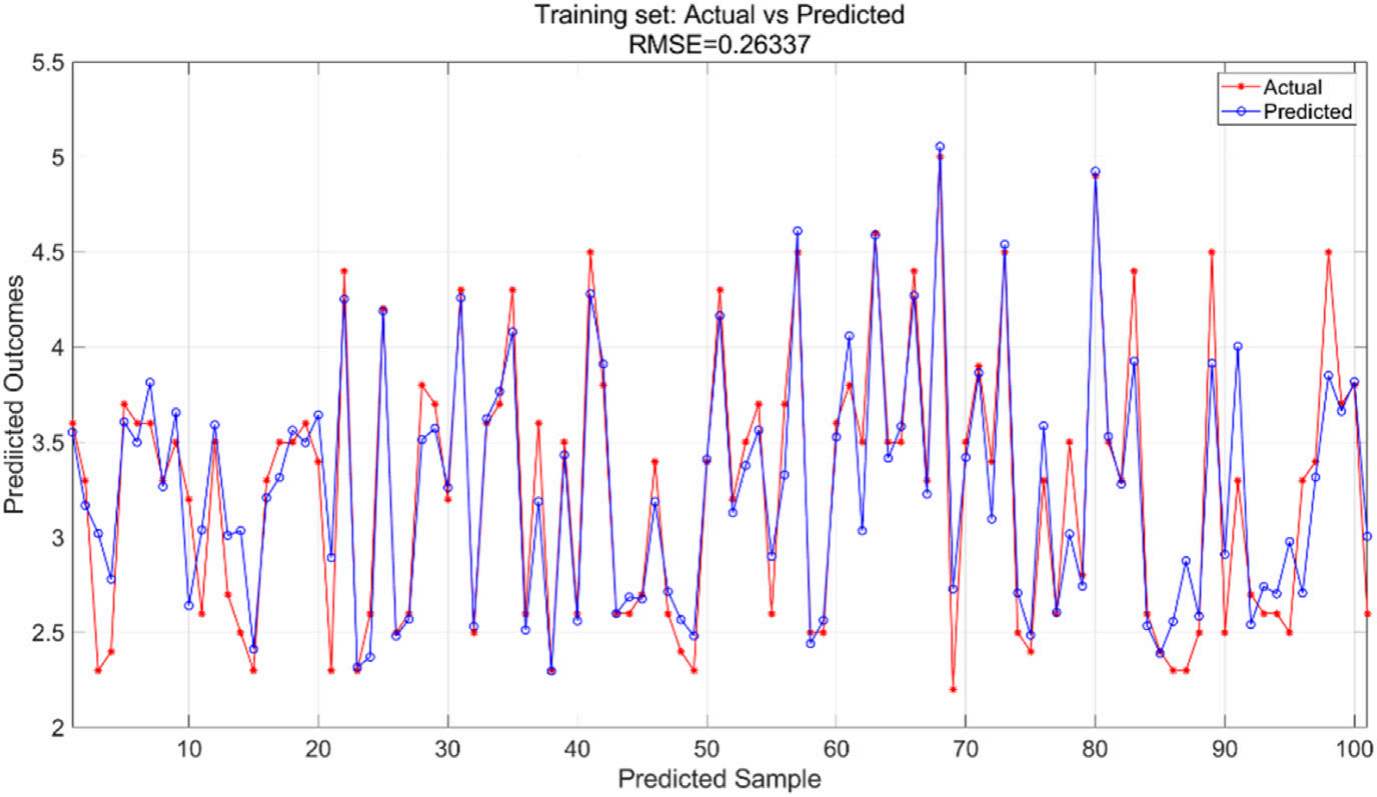
Training set training predicted and actual value results.

**FIGURE 16:**
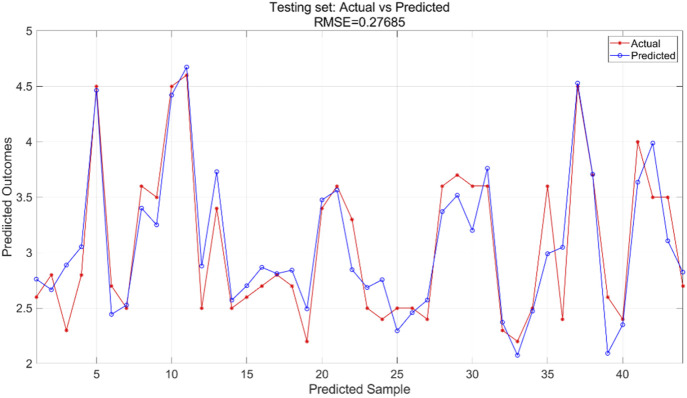
Test set prediction model predicted and actual value results.

All variable data are incorporated into the PSO-SVR model, with model parameters modified according to the requirements of particle swarm optimization. This includes adjustments to the learning factors of the particle swarm, the number of iterations, and the size of the particle swarm population based on the SVR. In the current experiment, the learning factors $c_{1}$ and $c_{2}$ are set to 1.7, the number of iterations for the particle swarm is 100, and the particle swarm population size is 10. The PSO-SVR model is trained using the training and test sets to predict outcomes, yielding iteration error results (Figure 14), training results for the training set (Figure 15), and prediction results for the test set (Figure 16).

The PSO algorithm illustrates how the model’s error evolves with the number of iterations. As shown in Figure 14, after approximately 10 iterations, the model’s error stabilizes within the range of 0.0265–0.027, indicating convergence of the optimization process. For the PSO-SVR model, the training dataset serves as the foundation for learning underlying patterns in the data. Figure 15 presents a comparison between predicted and actual values within the training set, achieving an RMSE of 0.26337, which reflects a good fit of the model to the training data. The test dataset, which was not exposed to the model during training, is utilized for validation purposes. Figure 16 displays the comparison between predicted and actual values in the test set, resulting in an RMSE of 0.27685. This value indicates that the PSO-SVR model maintains robust predictive performance on unseen data, with minimal degradation from the training performance, suggesting good generalization capabilities.

#### 3.2.5 Comparison of regression model results

**FIGURE 17:**
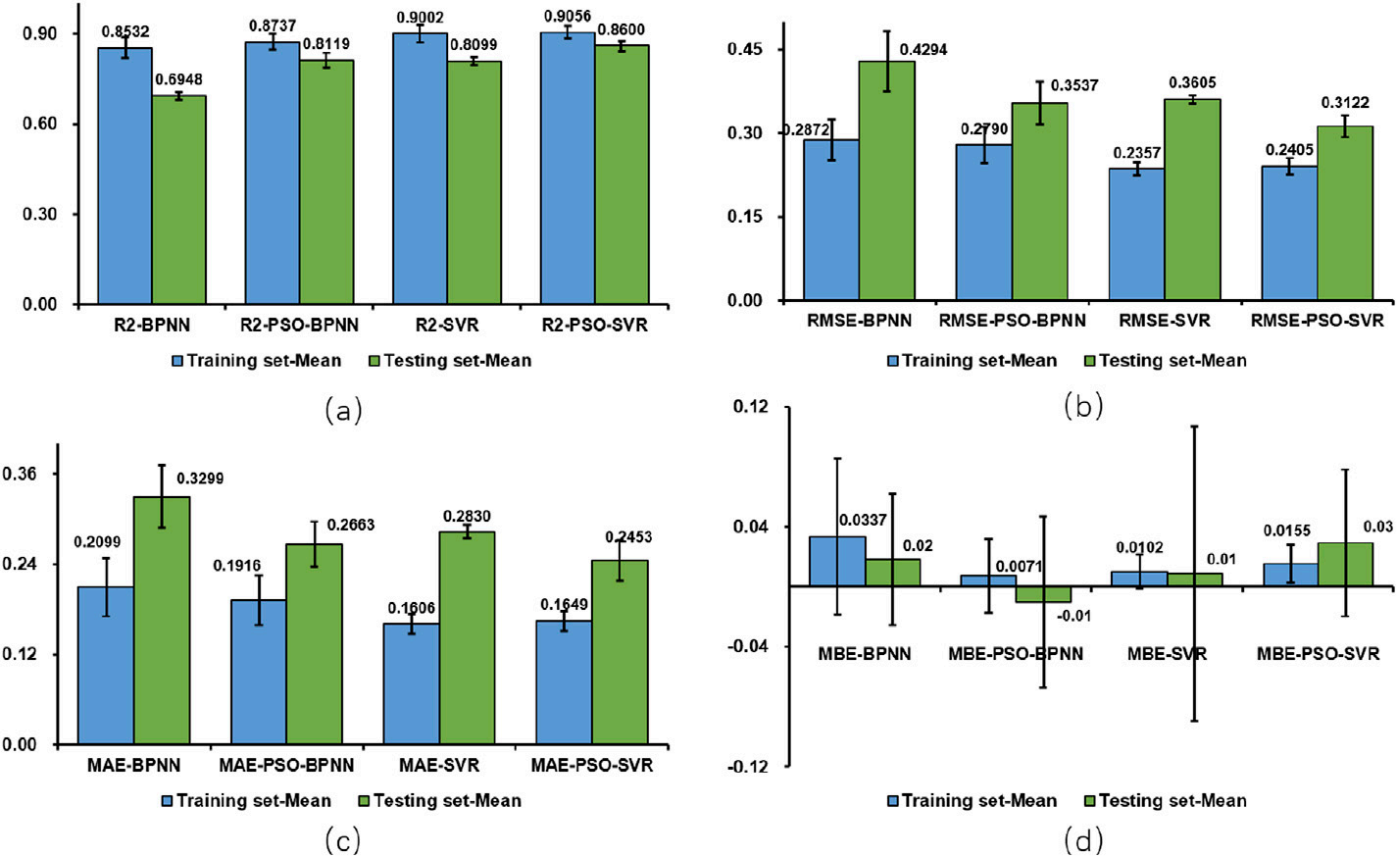
Comparison of regression models **(a)** *R* ^2^; **(b)** RMSE **(c)** MAE **(d)** MBE.

To analyze the performance of the machine learning models comprehensively, the performance metrics (*R* ^2^;, RMSE, MAE, and MBE) for both the test and training sets are illustrated visually in Figures 17a–d, while the findings for all test sets are summarized in Table 2 reveals significant disparities among the results of the four model test sets: BPNN, SVR, PSO-BPNN, and PSO-SVR. This indicates that the right shoulder anterior flexion sEMG and motion data collected in this experiment were modeled differently across the four machine learning frameworks. The PSO-SVR model exhibited the best fit, achieving a *R* ^2^; of 0.8600, followed by SVR with a *R* ^2^; of 0.8099, PSO-BPNN with a *R* ^2^; of 0.8119, and BPNN, which demonstrated the poorest fitting performance with a *R* ^2^; of 0.6948. The accuracy assessment metrics for the test sets of the four models are RMSE, MAE, and MBE. The PSO-SVR model exhibits an RMSE of 0.3122, an MAE of 0.2453, and an MBE of 0.0293, indicating superior prediction accuracy. The SVR model has an RMSE of 0.3605, an MAE of 0.2830, and an MBE of 0.0090, reflecting relatively high prediction accuracy. The PSO-BPNN model shows an RMSE of 0.3537, an MAE of 0.2663, and an MBE of 0.0099. The BPNN model records an RMSE of 0.4294, an MAE of 0.3299, and an MBE of 0.0184, indicating relatively high prediction accuracy.

**TABLE 2:** Results of the four model test set.

Model	$R^{2}$ (Mean ± SD)	RMSE (Mean ± SD)	MAE (Mean ± SD)	MBE (Mean ± SD)
BPNN	0.6948 ± 0.0133	0.4294 ± 0.0535	0.3299 ± 0.0411	0.0184 ± 0.0438
SVR	0.8099 ± 0.0129	0.3605 ± 0.0075	0.2830 ± 0.0088	0.0090 ± 0.0985
PSO-BPNN	0.8119 ± 0.0256	0.3537 ± 0.0379	0.2663 ± 0.0299	0.0099 ± 0.0571
PSO-SVR	0.8600 ± 0.0173	0.3122 ± 0.0201	0.2453 ± 0.0270	0.0293 ± 0.0490

Upon integrating the four evaluation metrics, it is evident that the PSO-SVR and SVR models outperform the PSO-BPNN and BPNN regression models. Furthermore, the PSO-SVR and PSO-BPNN predictive models demonstrate a marked superiority over the SVR and BPNN predictive models, indicating that the particle swarm optimization algorithm substantially improves the model’s overall efficacy, thereby facilitating more accurate predictions. To enhance the robustness of performance evaluation, we conducted multiple repeated experiments for each model and reported the performance metrics as mean ± standard deviation (SD). This allows assessment of both central tendency and variability. In addition, the consistent superiority of the PSO-SVR model across repeated trials suggests good generalizability and model stability, even under limited sample conditions. Although test-retest reliability and minimal detectable change (MDC) were not formally assessed, these will be the focus of future clinical studies. To assess whether the performance differences among the four regression models were statistically significant, we conducted repeated-measures one-way ANOVA on the cross-validated *R* ^2^; and RMSE values across folds. Post hoc pairwise comparisons were performed using Bonferroni correction to control for multiple comparisons. Results showed that the PSO-SVR model achieved significantly higher $R^{2}$ and lower RMSE compared to the other models (p<0.05) , confirming that its superior performance was not due to random variation. This statistical validation supports the robustness of our model selection. To assess the robustness of performance differences, 95% confidence intervals (CIs) were calculated for each model’s $R^{2}$ , RMSE, MAE, and MBE using cross-validation results. In addition, repeated-measures ANOVA with Bonferroni post hoc tests was applied to determine whether the observed differences were statistically significant. The PSO-SVR model showed significantly higher $R^{2}$ (95% CI: [0.8010, 0.8754]) compared to the other models (p<0.05) , confirming that its superior performance was not due to random variation.

## 4 Discussion

This work employs two prevalent machine learning regression prediction models, alongside regression prediction models utilizing particle swarm optimization: BPNN, SVR, PSO-BPNN, and PSO-SVR. In experiments involving shoulder forward flexion exercises of the right upper limb on 15 subjects, four regression models—BPNN, SVR, PSO-BPNN, and PSO-SVR—were evaluated for their fitting performance and accuracy. The model fitting performance and accuracy indicated that the coefficient of determination *R* ^2^; for PSO-SVR surpassing SVR, both of which exceeded the performance of PSO-BPNN and BPNN. Additionally, the accuracy of PSO-SVR and SVR was markedly superior to that of PSO-BPNN and BPNN. Furthermore, PSO-SVR and PSO-BPNN demonstrated significantly enhanced model performance and accuracy compared to SVR and BPNN, suggesting that the particle swarm optimization algorithm enhances the model’s global search capability, resulting in improved prediction accuracy and performance. The PSO-SVR and SVR models outperform the PSO-BPNN and BPNN models significantly. The performance enhancement of the particle swarm optimization-based algorithm, in comparison to the original algorithm, is primarily attributed to its capacity for global search within the parameter space to identify optimal model parameter configurations. This approach increases the likelihood of discovering the global optimal solution and prevents the traditional model from becoming trapped in local optima (Wang D. et al., 2018; Jensi and Jiji, 2016). Particle swarm optimization algorithms have been shown across various domains to explore the model’s structure and parameter space, identify the optimal model fit, and effectively elucidate the intricate relationships among data (Cui et al., 2017), thereby enhancing model performance, predictive accuracy, and generalization capability.

The performance of the SVR model in the context of the right upper extremity shoulder forward flexion exercise is markedly superior to that of the BPNN. This superiority is evident in the SVR model’s adeptness at managing small sample data by employing support vectors for data fitting and its resilience against outlier interference. Conversely, the BPNN is susceptible to overfitting with small sample sizes and may be adversely affected by outliers, leading to inadequate generalization capabilities and diminished performance.

The experimental findings in this paper indicate that the RMSE and MAE of the PSO-BPNN test set are inferior to those of the BPNN, signifying superior prediction accuracy for PSO-BPNN. Concurrently, the absolute value of the MBE for the PSO-BPNN test set exceeds that of the BPNN, primarily attributable to the PSO-BPNN model’s heightened sensitivity to outliers during the optimization process compared to the BPNN model, particularly in the presence of outliers or noisy data within the test set. The presence of outliers or noisy data in the test set disrupts the PSO-BPNN model during the fitting process, leading to a greater absolute value of MBE, whereas the BPNN model, being less susceptible to outliers, demonstrates superior performance in terms of MBE. The experiment’s outcome underscores the necessity of using many metrics during model selection and evaluation to thoroughly evaluate the model’s performance and flexibility. While our findings in healthy subjects provide a solid foundation for system development, we acknowledge that clinical application requires validation in the target population. Therefore, as the next step, we plan to conduct studies involving stroke patients to evaluate the system’s predictive performance, usability, and clinical responsiveness in real-world rehabilitation scenarios. Such investigations will be critical for translating this technology into individualized, patient-centered rehabilitation strategies.

The purpose of this study was focuses on patients with upper limb motor dysfunction following a clinical stroke, enabling rehabilitation therapists to utilize equipment for gathering upper limb movement data and employing suitable models to forecast muscle strength outcomes, thereby offering a basis for developing personalized and precise rehabilitation programs. Improve the model’s overall performance, hence facilitating more accurate predictions of outcomes.

This study has several limitations that should be acknowledged.

First, the participant cohort consisted exclusively of healthy adults aged 20–28 years. Although this controlled demographic enabled standardized signal acquisition and technical feasibility analysis, it limits the generalizability of our findings. Stroke survivors commonly display altered muscle activation patterns, reduced motor unit synchronization, increased co-contraction, and abnormal joint kinematics due to neuromuscular impairments. These distinctions may affect both the distribution of sEMG and IMU features and the generalization ability of machine learning models trained solely on healthy data. Our future work will involve clinical validation in a wider age range of stroke populations and the development of relevant databases (Dhahbi et al., 2014).

Second, this study employed a single-joint, unidirectional shoulder flexion task, which—while technically controllable—fails to reflect the multidimensional demands of upper-limb functional tasks. Stroke rehabilitation often involves multi-joint coordination, varied movement directions, and goal-oriented behaviors like reach-to-grasp, object transport, and bilateral task execution. To capture these dynamics, future studies should incorporate more functionally relevant movement paradigms and extract kinematic quality metrics such as trajectory smoothness, joint synchronization, and compensatory trunk motions using IMU-derived features or video-based motion tracking.

Third, we used Manual Muscle Testing (MMT) scores as the ground truth labels for training regression models. Although MMT is widely accepted in clinical practice for its simplicity and availability, it remains a subjective, ordinal-scale assessment. Treating MMT scores as continuous variables introduces approximation error: the coarse granularity and inter-rater subjectivity may inject noise into model training and affect interpretability of outcome metrics such as RMSE and $R^{2}$ . Despite these limitations, MMT offers a practical and clinically accessible starting point. In future work, we plan to explore ordinal regression models or hybrid structures that respect the ordered nature of MMT scores. Moreover, transitioning to more objective and continuous outcome standards, such as force sensors or dynamometry, may further enhance model precision and validity.

Fourth, while this study emphasized model accuracy metrics, we acknowledge the importance of validating the system’s reliability and clinical sensitivity. Future research will include test-retest reliability analysis using established metrics such as the intraclass correlation coefficient (ICC), standard error of measurement (SEM), and minimal detectable change (MDC), especially in longitudinal stroke rehabilitation trials (Čular et al., 2021). Additionally, external responsiveness—the ability of the system to detect meaningful physiological or functional change over time—must be demonstrated. We plan to evaluate responsiveness following standardized frameworks (Ardigò et al., 2020), including pre-post intervention testing and analysis of effect size or standardized response mean (SRM), to assess the system’s utility in real-world clinical recovery tracking.

## 5 Conclusion

This research proposes a muscle strength rating system for upper extremity rehabilitation, utilizing movement velocity and offset angle data obtained from sEMG and MPU6050. The approach tackles the existing issue of activity constraints resulting from an exclusive dependence on sEMG for movement recognition or classification. The analysis focuses on the muscle strength grade of the right upper limb’s shoulder forward flexion movement, utilizing sEMG and motion capture technologies to develop a machine learning regression model for predicting muscle strength grading. Experiments have demonstrated that the upper limb muscle strength evaluation model, which is based on sEMG and joint motion information presented in this paper, can serve as an effective reference for shoulder forward flexion exercises and rehabilitation training, offering theoretical technical support for therapists in designing personalized rehabilitation programs.

## Data Availability

The raw data supporting the conclusions of this article will be made available by the authors, without undue reservation.
